# Survival of midbrain dopamine neurons depends on the Bcl2 factor Mcl1

**DOI:** 10.1038/s41420-018-0125-7

**Published:** 2018-11-21

**Authors:** Edward J. Robinson, Sebastian P. Aguiar, Willemieke M. Kouwenhoven, Dorinde S. Starmans, Lars von Oerthel, Marten P. Smidt, Lars P. van der Heide

**Affiliations:** Swammerdam Institute for Life Sciences, University of Amsterdam, Science Park 904, 1098 XH Amsterdam, The Netherlands

## Abstract

Mitochondria-dependent apoptosis plays an important role in the embryonic development of the midbrain dopaminergic system as well as in Parkinson’s disease. Central to mitochondria-dependent apoptosis is the Bcl2 family of apoptosis-regulating proteins. However, it was unclear which Bcl2 proteins are important for the survival of dopaminergic neurons. Here, we identify Mcl1 as a critical Bcl2 pro-survival factor in midbrain dopaminergic neurons. Using a chemical biology approach to inhibit various components of the apoptotic machinery in the dopaminergic MN9D cell line or the control neuroblastoma N2A cell line, we find that functional inhibition of Mcl1 with the high affinity small molecule inhibitor UMI-77 results in a rapid and dose-dependent loss of viability, selectively in dopaminergic cells. In-depth analysis of the apoptotic signaling pathway reveals that chemical inhibition of Mcl1 results in the activation of Bax, activation of cleaved caspase-3 and finally cell death. The dependence of mouse dopaminergic midbrain neurons on Mcl1 was confirmed using ex vivo slice cultures from Pitx3GFP/+ and wildtype mice. In mouse dopaminergic midbrain neurons positive for the midbrain dopaminergic marker Pitx3, or tyrosine hydroxylase, UMI-77 treatment caused a dramatic increase in cleaved caspase 3, indicating that Mcl1 activity is required for basal neuronal survival. Overall, our results suggest that Mcl1 is of critical importance to dopaminergic neurons and is a weak link in the chain controlling cellular survival. Boosting the pro-survival function of Mcl1 should be pursued as a therapeutic approach to augment the resilience of midbrain dopaminergic neurons to apoptotic stress in Parkinson’s disease.

## Introduction

Parkinson’s disease (PD) is the second most common neurodegenerative disease after Alzheimer’s disease^1^. Its clinical symptoms arise due to the progressive death of dopamine neurons in the substantia nigra pars compacta (SNpc) resulting in the loss of dopaminergic input to the striatum. There is no cure for Parkinson’s disease and current palliative treatments are mostly based on supplementation with L-DOPA, the blood–brain-barrier permeable precursor for dopamine. At the onset of clinical symptoms it is estimated that more than 60% of the dopamine neurons have died^1^. Attenuating the loss of dopamine neurons would be a potential breakthrough in the treatment of PD. Of the different forms of cell death it is suggested that mitochondria-dependent apoptosis plays a major role in the loss of dopaminergic neurons in PD^2,3^, although the exact mechanism and signaling components remain obscure.

Mitochondria-dependent apoptosis is executed by the formation of a proteolipid pore in the outer mitochondrial membrane by oligomers of Bax and/or Bak^4^. The formation of these proteo-lipid pores allows components such as Cytochrome *C*, Apoptosis Inducing Factor (AIF), and Endonuclease G to leak out from the mitochondrial intermembrane space into the cytosol. Following mitochondrial outer membrane permeabilization (MOMP), mitochondrial contents spill into the cytosol to activate caspases resulting in the apoptotic phenotype^4^.

The apoptotic effector proteins Bax and Bak are the executioners of a precisely calibrated network of a large cast of pro and anti-apoptotic Bcl2 family proteins^4^. The subfamily of anti-apoptotic Bcl2 proteins consists of Bcl2, Bcl-w, A1, Bcl-xl, and Mcl1. Whereas the pro-apoptotic Bcl2 family consists of the effector proteins Bax, Bak, and Bok and the BH3-only proteins, Noxa, Puma, Bim, Bad, Bid, Bmf, Hrk, Bik, and Bnip3.

We have previously suggested that development of the midbrain dopaminergic system depends on a complex interplay between factors controlling transcription, growth, and survival^2^. These factors control life and death of each individual dopamine neuron through the regulation of its unique set of Bcl2 factors. Recalibrating the Bcl2 composition in favor of cellular survival may strengthen dopaminergic neurons against stressful events which would normally result in apoptosis.

In this study, we set out to characterize the apoptotic machinery that controls dopaminergic cell death and identify the pro-survival Bcl2 family factor which is the weakest link for cellular survival. Previous data shows that overexpression of ectopic Bcl2 in dopamine neurons results in a higher number of dopamine neurons in mice after birth and protects against toxin-induced degeneration^5^. However, recent microarray experiments suggest that the relative expression levels of endogenous Mcl1 are higher in dopaminergic neurons as compared to Bcl2^6^. Possibly, Mcl1 is of greater importance in mediating survival than Bcl2.

By leveraging the toolbox of high affinity small molecule inhibitors designed to induce apoptosis in cancer cells, we screened which inhibitors preferentially resulted in death of dopaminergic vs. non-dopaminergic neuronal cells. Our screen identified Mcl1 as crucial for dopaminergic neuronal survival, whereas Bcl2 and Bcl-xL were of crucial importance in the non-dopaminergic neuronal cell line. In-depth analysis of the apoptotic pathway confirmed that Mcl1 indeed controls apoptosis via the canonical Bax-Caspase-3 pathway. Additionally, overexpression of Mcl1 greatly augmented the resilience against the DNA-damage inducing apoptotic stressor etoposide in dopaminergic cells. Testing the resilience of Mcl1 in ex vivo mouse midbrain slices from both Pitx3GFP/+ and wildtype (WT) mice confirmed the importance of Mcl1 as a crucial Bcl2 factor in dopaminergic survival. Targeting the regulatory pathways controlling Mcl1 protein levels and function may therefore provide a novel therapeutic approach to strengthen dopaminergic neurons against apoptotic stress in Parkinson’s disease.

## Results

### Mcl1 inhibitors preferentially induce caspase-3 activity in dopaminergic cells

**Fig. 1:**
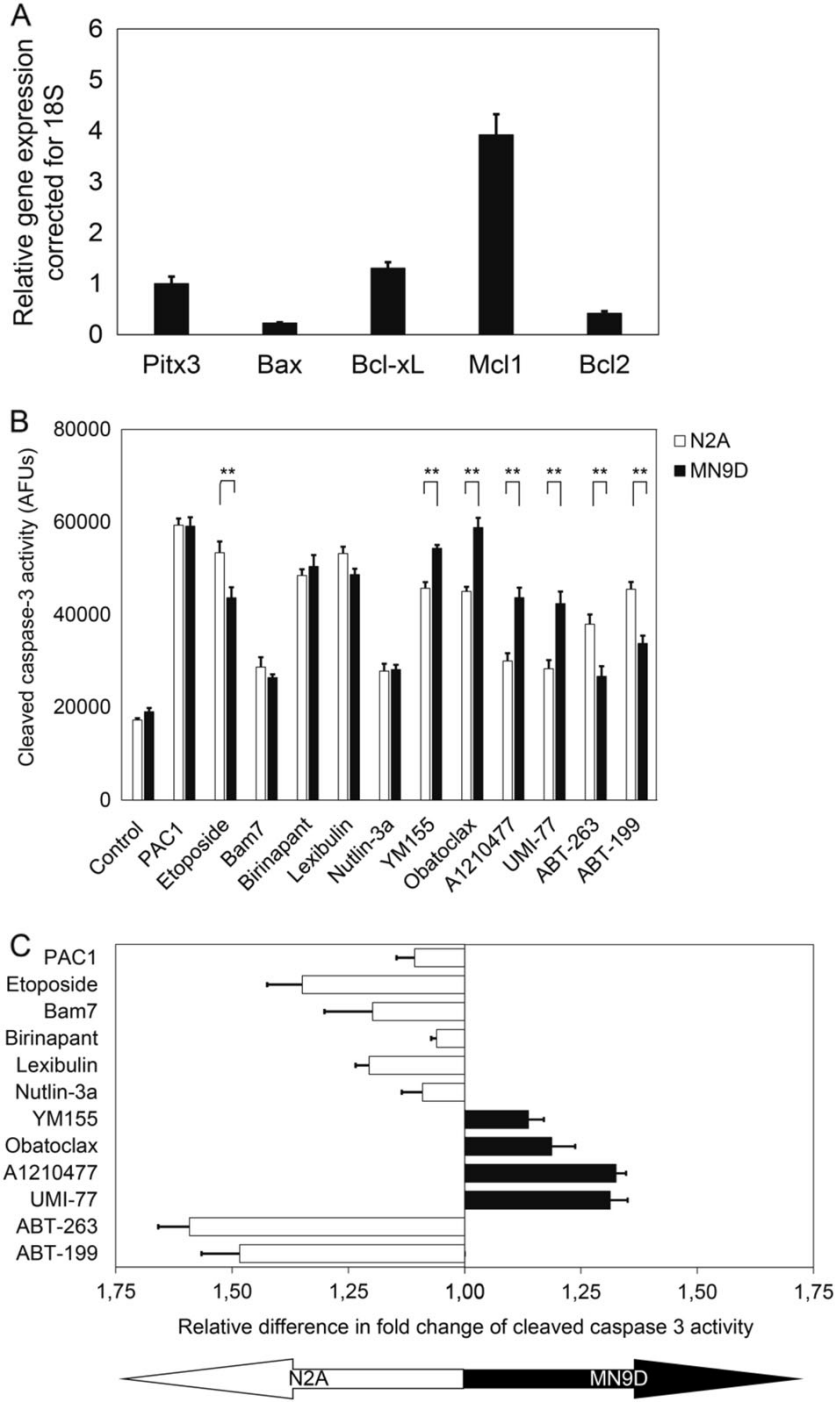
**a**. Mcl1 is abundantly expressed in mouse midbrain dopaminergic neurons. 2-day old Pitx3/GFP mice were killed, brains were isolated, and dissociated with papain followed by FACsorting. GFP-positive cells were collected and RNA was purified before RT-qPCR analysis with primers designed to specifically detect indicated transcripts. **b** Vulnerability of MN9D and N2A cell lines to a small molecule screen of apoptosis-inducing compounds. MN9D and N2A cells were incubated for 16 h at concentrations indicated. Cells were then treated with 0.1% Triton X-100 for 30 min, followed by 30 min incubation in DEVD-AMC (10 µM), a fluorogenic caspase 3 substrate. Fluorescence intensity was measured using a BioTek Synergy H1 plate reader. **c** Relative difference in fold change of cleaved caspase 3 activity of MN9D vs N2A cells. (*n* = 3, mean ± SD. Unpaired Student’s *t*-test, *p*-value: **P < 0.01)

Previous studies have suggested an important role for Bcl2 itself in controlling dopaminergic survival, whereas a role for Mcl1 has not been described^5^. However, micro-array data^6^ and RT-qPCR data performed on sorted dopamine midbrain neurons from Pitx3GFP/+ heterozygous mice suggest that Mcl1 is one of the most highly expressed pro-survival Bcl2 factors in dopamine neurons. During mouse embryogenesis (not shown) as well as at P2, *Mcl1* and *Bcl-xL* can abundantly be detected in dopaminergic neurons, whereas *Bax* and *Bcl2* are less present (Fig. 1a). To test the relative importance of Bcl2, Bcl-xL, and Mcl1, we used a small molecule chemical screen coupled with readouts for apoptotic cell death in two cell lines, the dopaminergic MN9D and the more commonly used non-dopaminergic neuroblastoma N2A cell line. The MN9D cell line was created by fusing microdissected E14 mouse rostral mesencephalic tegmentum cells from the approximate SNc/VTA with a neuroblastoma cell line (N18TG2) of neural crest origin. Unlike another commonly used DAergic cell line, PC12 (derived from an adrenal medullary tumor), MN9D dopamine content can be depleted by low dose MPP+, which is often used as a tool to induce PD like symptoms in rodents. MN9D cells are commonly used to test mechanisms and drug candidates relevant to PD. By contrasting the degree of cell death in MN9Ds vs. N2As, we aimed to identify weak links in survival that are specific to dopaminergic cells. To probe the relative weak links in apoptotic signaling of MN9Ds and N2As, the cells were dosed overnight with a panel of small molecule chemical inhibitors (Table 1, Fig. 1b), and their relative cell-type specificity was calculated (Fig. 1c). PAC1 is a caspase-3 activator, and served as a positive control (while the signal was high, there was no significant difference between the cell lines). The small molecules ABT-263, ABT-199 preferentially induced caspase-3 activity in N2A cells as compared to MN9D cells which suggests a more prominent role for Bcl2 and Bcl-xL as compared to Mcl1^10^. High affinity inhibitors UMI-77, A1210477, and Obatoclax (which inhibit Mcl1 protein function)^8^, and YM155 (transcriptional inhibition of Mcl1 expression) preferentially induced caspase-3 activity in MN9D cells, which suggests that Mcl1 is of greater importance in dopaminergic cells as compared to N2A cells.

**Table 1:** Small molecule inhibitors and stressors used in cleaved caspase 3 activity assay to determine the vulnerability of MN9D and N2A cells.

Compound	Works as	Concentration (µM)
PAC1	Caspase-3 activator	10
Etoposide	Topoisomerase inhibitor	5
Bam7	Bax activator	10
Birinapant	cIAP1 inhibitor /Birc2 inhibitor	10
Lexibulin	Microtubule inhibitor	1
Nutlin-3a	p53 inhibitor	10
YM155	Mcl1 inhibitor /Survivin inhibitor	10
Obatoclax	Mcl1 inhibitor /Bcl2 inhibitor	10
A1210477	Mcl1 inhibitor	10
UMI-77	Mcl1 inhibitor	10
ABT-263	Bcl-xL inhibitor	10
ABT-199	Bcl2 inhibitor	10

### Mcl1 but not Bcl-xL is required for dopaminergic cell survival

**Fig. 2:**
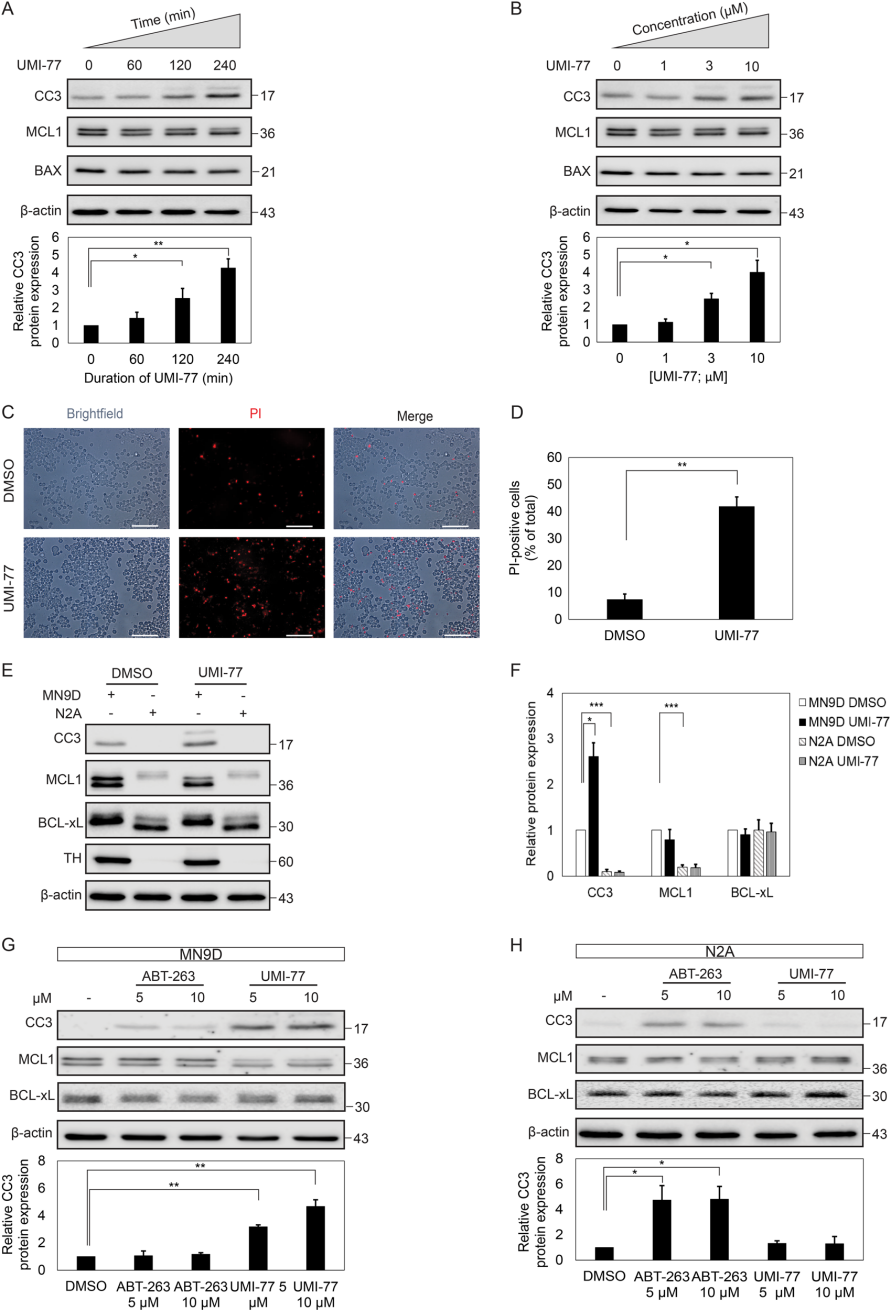
Mcl1 inhibition leads to cell death of dopaminergic cells. **a** MN9D cells were grown and subsequently treated with 10 µM UMI-77 for 60, 120, or 240 min and subsequently analyzed by western blot for the amount of cleaved caspase 3. Mcl1, Bax, and actin were probed as controls. The amount of cleaved caspase 3 was normalized for the amount of actin and depicted in the bar graph. Quantification of the western blot shows that UMI-77 time-dependently increases the amount of cleaved caspase 3. **b** MN9D cells were grown as in **a**, but treated with 1, 3, or 10 µM of UMI-77 before analysis on western blot and quantification. Again amount of cleaved caspase 3 was corrected for the amount of actin. **c** Cells were grown as in A and treated with 10 µM of UMI-77 for 4 h and subsequently treated with propidium iodide to visualize permeant cells. Scale bar represents 50 µm. **d** Positive cells were quantified and represented as relative amount compared to total amount of cells. **e** Dopaminergic MN9D cells and non-dopaminergic cells were both treated with UMI-77 for 4 h and subsequently analyzed by western blot for the amount of cleaved caspase 3, Mcl1, Bcl-xl, Th, and actin. The actin corrected relative amount of cleaved caspase 3 is indicated in the bar graph. Additionally, levels of Mcl1 and Bcl1-xL were quantified and corrected for actin (**f**)**. g**, **h** MN9D and N2A cells were treated with ABT-263 or UMI-77 at 5 and 10 µM for 4 h and subsequently analyzed by western blotting for the amount of cleaved caspase 3, Mcl1, Bcl-xL, and actin. Relative levels of cleaved caspase 3 were corrected for actin and depicted in the bar graphs. Significance was determined with a Student’s -test, *p* value: *p < 0.05, **p < 0.01, ***p < 0.005 (*n* = 3)

A time-curve experiment revealed that UMI-77 application to MN9D cells results in a significant increase in cleaved caspase-3 within 2–4 h (Fig. 2a). A dose-response curve revealed that at least 3 µM of UMI-77 is enough to induce a significant increase in cleaved caspase-3 (Fig. 2b). To assess if the increase in cleaved caspase-3 corresponds with the induction of cell death, MN9D cells were treated with propidium iodide (PI) after Mcl1 inhibition with UMI-77 (Fig. 2c). Live cells are impermeable to PI, staining only the DNA of dying (late apoptotic) or dead cells. Indeed, the amount of PI-positive cells dramatically increased after UMI-77-dependent Mcl1 inhibition, confirming that UMI-77 induces cell death in MN9D cells (Fig. 2d).

Since UMI-77 had less effect on N2A cells as compared to MN9D cells (Fig. 1b, c), we compared several parameters between these cell types in response to UMI-77 application. N2A cells did not show an increase in cleaved caspase-3 in response to UMI-77 application whereas MN9D cells did (Fig. 2e). Probing for Mcl1, the primary target of UMI-77, revealed that N2A cells express lower levels of Mcl1 as compared to MN9D cells, whereas Bcl-xL, the primary target of ABT-263^10^, was expressed at comparable levels (Fig. 2e). Probing for Th confirmed the dopaminergic phenotype of MN9D cells and the non-dopaminergic character of N2A cells as Th is not expressed in N2A cells. Th levels were not altered by UMI-77 incubation. To directly compare the effect of Mcl1 or Bcl-xL inhibition on cell death in N2A and MN9D cells, we incubated both cell types with either UMI-77, to inhibit Mcl1, or ABT-263, to inhibit Bcl-xL. UMI-77 induced a clear increase in cleaved caspase-3, but failed to do so in N2A cells (Fig. 2g, h). ABT-263 showed the inverse, as it did not result in a robust induction in cleaved caspase-3 in MN9D cells, but did show a significant increase in N2A cell death, revealing cell type-specific effects and the reliance of MN9D cells on Mcl1 (Fig. 2g, h).

### Mcl1 prevents Bax-mediated canonical mitochondria-dependent apoptosis

**Fig. 3:**
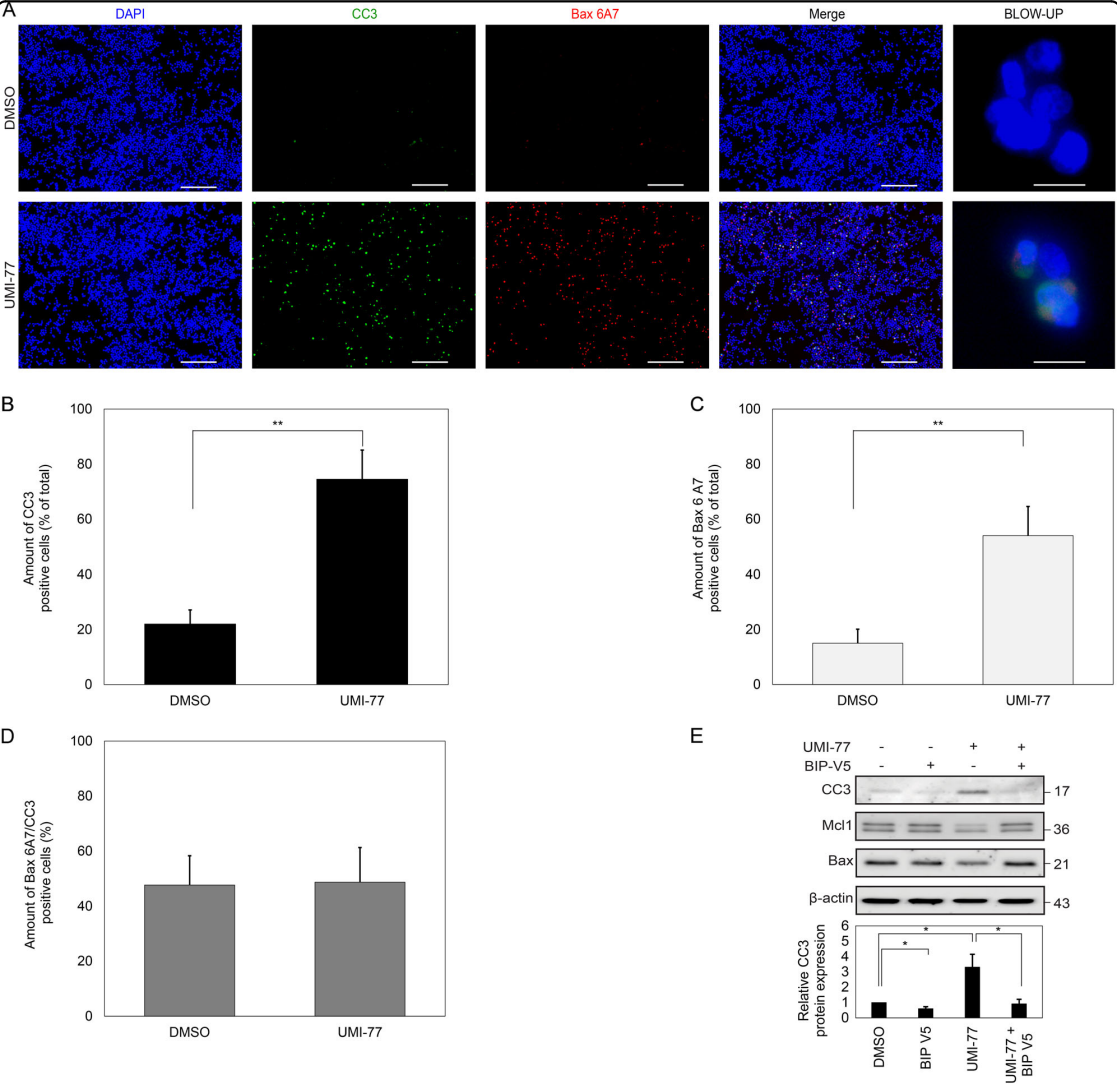
UMI-77-dependent cell death requires Bax activation. **a** MN9D cells were treated with UMI-77 for 4 h followed by PFA fixation and staining with indicated antibodies. Scale bar represents 50 µm. Blow-up shows cell triple positive for DAPI, cleaved caspase 3 and Bax6A7. Scale bar represents 5 µm. **b** Bar graphs indicate percentage of cleaved caspase 3 positive cells relative to total cells, percentage of Bax6A7 positive cells relative to total cells (**c**) and percentage of cleaved caspase 3 cells which are also positive for Bax6A7 (**d**). **e** Western blot of cells pre-incubated with BIP-V5 before UMI-77 application for 4 h. Western was detected with various antibodies as indicated. Significance was determined with a Student’s *t*-test, *p* value: *p < 0.05, **p < 0.01 (*n* = 3)

Inhibition of the anti-apoptotic function of Mcl1 results in an increase in cleaved caspase-3 as well as an increase in the permeability of the cell to PI, strongly indicting a mitochondrial-dependent cell death pathway (Fig. 2). Canonical mitochondrial-dependent intrinsic apoptosis requires the activation of Bax and/or Bak^4^. Upon activation, Bax undergoes a conformational change which exposes its N-terminal domain. Visualization of this exposed domain with a conformation-specific epitope antibody revealed that its staining strongly correlated with activation of caspase 3 (Fig. 3a, b). Activated Bax and cleaved caspase 3 show a significant overlap and suggests a common pathway (Fig. 3c). The Bax6A7 positive, cleaved caspase 3 negative cells and the Bax6A7, cleaved caspase-3 positive cells may represent phases of cell death which are either too early or too late for both markers. Since canonical apoptosis depends on Bax activation followed by MOMP and subsequent caspase activation, inhibition of Bax activation should block apoptosis. BIP-V5 is a 5-amino acid peptide designed to inhibit Bax activation. Pre-incubation of dopaminergic MN9D cells with BIP-V5 almost completely prevented the increase in cleaved caspase-3 upon the inhibition of Mcl1 with UMI-77, confirming canonical mitochondrial-dependent apoptosis (Fig. 3e). Interestingly, the fact that the inhibition of Bax with a small peptide is able to prevent cell death supports the strategy that dopaminergic neurons can be strengthened by strategically boosting weak links in the pathways controlling survival – a finding in concordance with a previous report in which 6-OHDA induced nigral cell loss could be prevented by BIP-V5^11^.

### Overexpression of Mcl1 protects against apoptotic stress

**Fig. 4:**
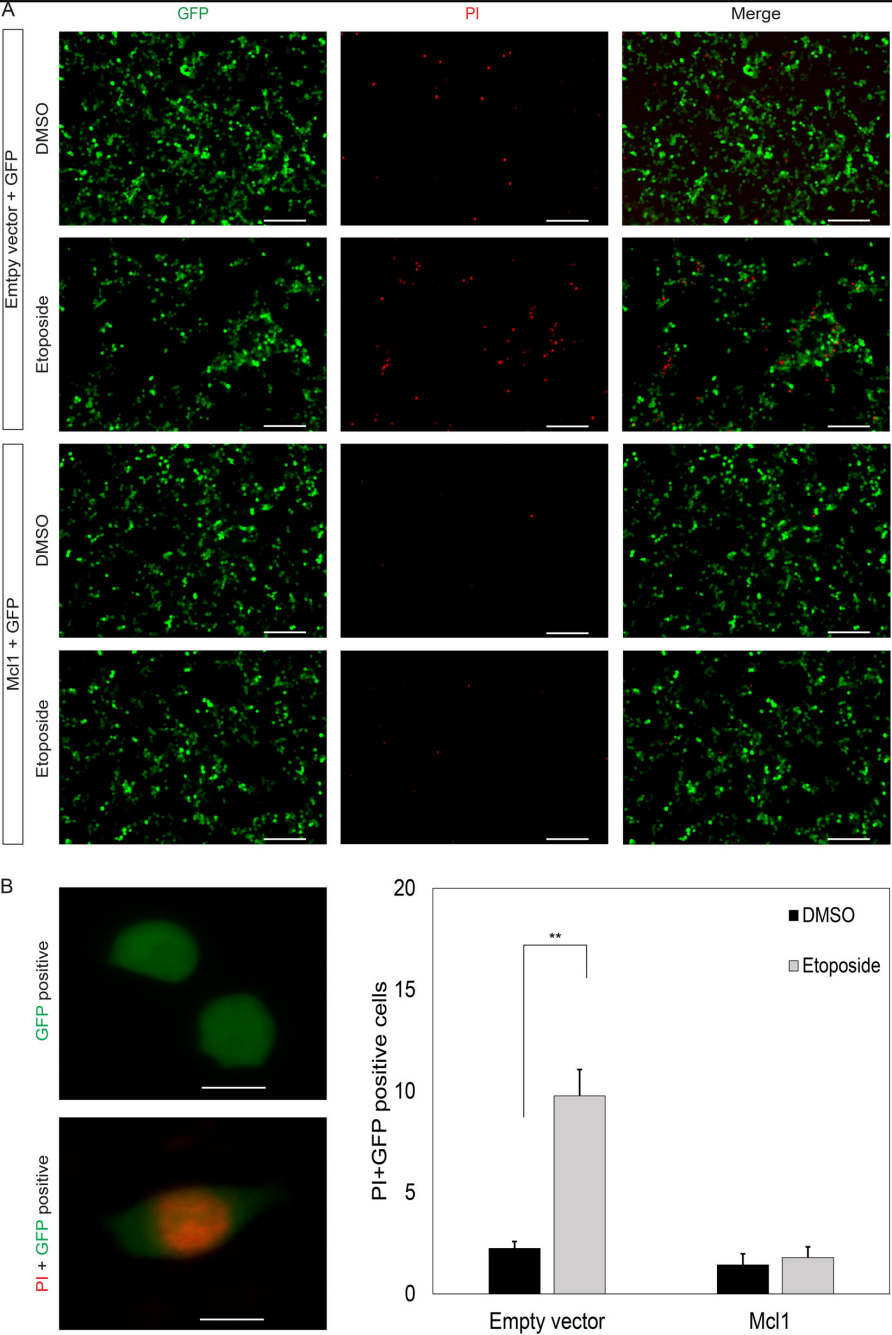
Overexpression of Mcl1 protects against apoptotic stress. **a** Immunofluorescent staining of cells transfected with empty vector or plasmid encoding for Mcl1. Cell were co-transfected with GFP as a marker for transfected cells and subsequently treated with etoposide for 8 h before treatment with PI. Scale bar represents 50 µm**. b** Left: blow up of representative example of GFP positive cell and dual GFP and PI positive cell. Scale bar represents 5 µm. Right: quantification of results seen in **a**. The amount of cleaved caspase 3 positive cells was determined relative to total GFP positive cells. Significance was determined with a Student’s *t*-test, *p* value: **p < 0.01 (*n* = 3)

Previously, it has been shown that ectopic overexpression of Bcl2 in dopaminergic neurons results in more dopaminergic neurons at birth as well as increased resistance to cell death-inducing stress^5^. Additionally, ectopic expression of the kinases SGK and AKT/PKB have been shown to exert a protective effect on dopamine neurons^12,13^. Interestingly, these kinases target various Bcl2 factors such as the BH3-only factor Bad^14^. More recently, the E3 ubiquitin ligase Parkin has been linked to the Bcl2 system, because Bax was described as one of the targets of Parkin, suggesting that mutations in Parkin would indirectly increase the levels of pro-apoptotic Bax^15^. Since Mcl1 inhibition induces apoptosis more vigorously than the inhibition of other anti-apoptotic Bcl2 factors, we overexpressed Mcl1 together with GFP in MN9D cells and subsequently treated the cells with the apoptotic stressor etoposide (Fig. 4a). Quantification of PI- and GFP-positive cells reveal that under baseline conditions Mcl1 overexpression already reduces the frequency of cell death, although not significantly, suggesting pro-survival effects. Etoposide treatment dramatically increases the amount of PI positive cells in controls, whereas Mcl1 greatly attenuates the amount of dead cells, confirming its protective effects against apoptotic stressors (Fig. 4b).

### Mcl1 activity is required for survival of dopaminergic neurons ex vivo

**Fig. 5:**
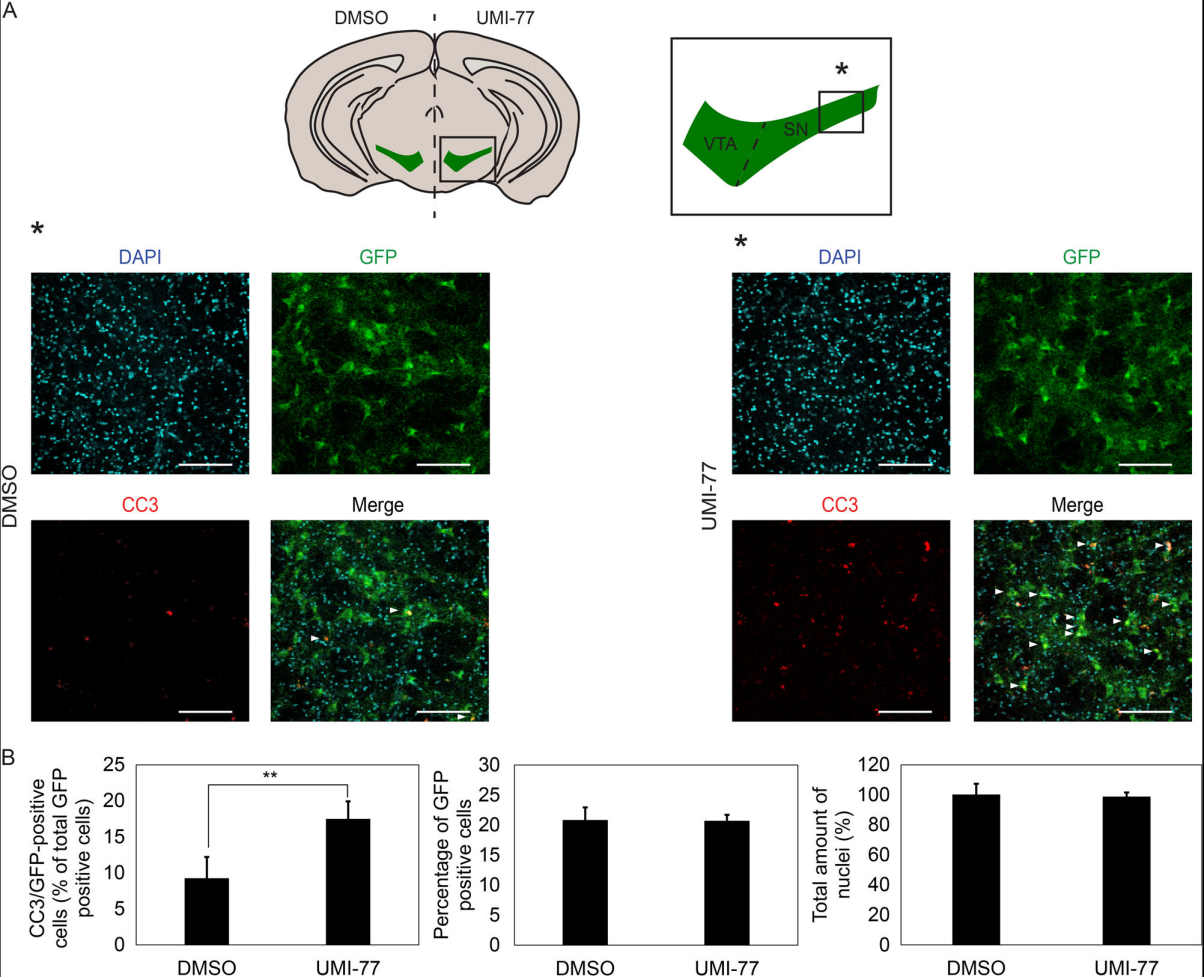
Mcl1 is required for dopaminergic survival ex vivo. **a** Brains from adult Pitx3/GFP heterozygous mice were sliced, hemispheres were separated and either incubated overnight with vehicle or UMI-77 before fixation with PFA and staining for GFP, cleaved caspase 3 and total nuclei. Hemispheres were matched so that every treated hemisphere had its complementary hemisphere as control. After staining results were visualized and quantified. Data as indicated was obtained from the area indicated in the red square. Scale bar represents 100 µm. **b** Quantification of total amount of DAPI positive cells, GFP-positive cells, and cleaved caspase 3 and GFP-positive cells. **p < 0.01 (*n* = 7) as determined with a Student’s *t*-test

**Fig. 6:**
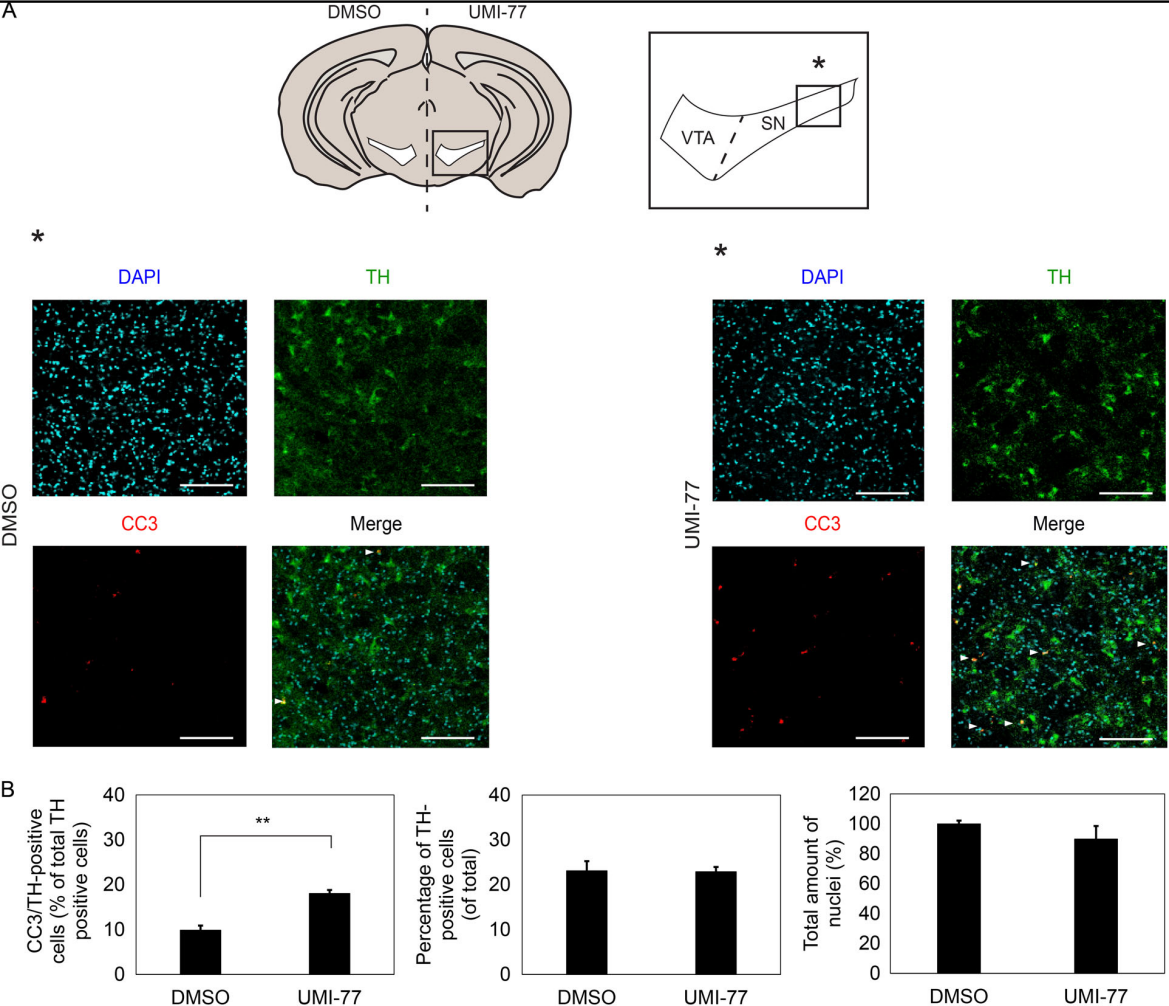
Mcl1 is required for dopaminergic survival ex vivo. **a** Brains from adult Bl6 mice were sliced, hemispheres were separated and either incubated overnight with vehicle or UMI-77 before fixation with PFA and staining for Th, cleaved caspase 3 and total nuclei. Hemispheres were matched so that every treated hemisphere had its complementary hemisphere as control. After staining results were visualized and quantified. Data as indicated were obtained from the area indicated in the red square. Scale bar represents 100 µm. **b** Quantification of total amount of DAPI positive cells, Th positive cells, and cleaved caspase 3 and Th positive cells. **p < 0.01 (*n* = 4) as determined with a Student’s *t*-test

To expand our findings obtained in MN9D and N2A cells and to establish whether Mcl1 is of crucial importance for the survival of dopaminergic neurons in the mouse midbrain, we made use of ex vivo brain slice cultures using heterozygous Pitx3GFP/+mice^9^ These mice develop normally and express GFP in every dopaminergic midbrain neuron. Mice were killed and their brains were sliced into coronal sections. Left and right hemisphere were split. One hemisphere was treated with UMI-77 to inhibit Mcl1 function, whereas the counter hemisphere served as its control (Fig. 5a). After an overnight incubation, slices were fixed and stained with DAPI, as well as for GFP and cleaved caspase-3 (Fig. 5a). Using confocal microscopy, a representative part of *substantia nigra* was selected and images were quantified using semi-automated counting in ImageJ. Images were scored for the total amount of cells using DAPI, relative amount of GFP positive cells and relative amount of GFP and cleaved caspase-3 positive cells. After quantifying all DAPI positive nuclei, no significant reduction was observed between the control and UMI-77 treated slices (*n* = 7) (Fig. 5b). About 20% of the total amount of cells were GFP positive, again no difference was observed between conditions, indicating that no obvious cell loss is observed after overnight treatment with UMI-77. Quantification of the number of GFP cells that were also positive for cleaved caspase-3 showed that about 10 percent of the GFP-positive neurons were positive, suggesting that the slicing procedure and culture conditions are harsh for the intrinsically vulnerable dopaminergic neurons. Possibly, the elevated mitochondrial demands of dopaminergic neurons as well as the large size of their axonal arborization as compared to other neurons contribute to the high amount of basal cell death^16^. Quantification of the amount of cleaved caspase-3 positive cells after UMI-77 treatment shows a doubling of the amount of positive cells as compared to control (*n* = 7) (Fig. 5b). The increase in the amount of cleaved caspase 3 levels, supports an essential role for Mcl1 as an active survival factor in dopaminergic neurons. Since Pitx3GFP/ + heterozygous mice only have one copy of *Pitx3* we repeated the experiment in mice containing Wt amounts of Pitx3 to exclude confounding effects caused by lower Pitx3 levels. Instead of GFP we stained Bl6 mice brain slices with Th as a marker for dopaminergic neurons (Fig. 6a). As for the Pitx3GFP/+ mice no significant reduction was observed in the total amount of DAPI positive cells (Fig. 6b). Quantification of the total amount of Th positive neurons indicates that there is also no loss after UMI-77 treatment as is observed for GFP in the Pitx3GFP/+mouse (Fig. 6b). Quantification of the amount of cleaved caspase 3-positive Th positive neurons indicates that about 10 percent are positive for cleaved caspase 3. UMI-77 increases the amount of cleaved caspase 3 positive neurons to slightly under 20 percent (Fig. 6b). Results in both Pitx3GFP/+P as well as in Wt BL6 mice suggest that UMI-77 increases basal cleaved caspase 3 activity, under the apparently harsh culture conditions about two-fold. These results support the data obtained in vitro, suggesting Mcl1 is of crucial importance for the survival of dopaminergic neurons.

## Discussion

Coordinated neuronal cell death in the mesencephalic dopaminergic system is crucial for proper embryonic development, whereas aberrant cell death in this system with age is the ultimate cause for the clinical symptoms observed in PD^1,2^. Mechanistically, the form of cell death implicated in development and PD is mitochondrial-dependent apoptosis which is controlled by various pro- and anti-apoptotic Bcl2 proteins^2,3^. However, the crucial and most essential Bcl2 factors have not been identified.

Here, by comparing non-dopaminergic to dopaminergic cells, using an in vitro and ex vivo approach we have pinpointed the Bcl2 factor Mcl1 as a weak link in the system actively controlling neuronal survival and reveal a therapeutic target to enhance the resilience of this vulnerable population of cells.

### Mcl1 as a dopaminergic survival factor

Historically, Mcl1 is the second identified pro-survival Bcl2 factor after Bcl2 itself^17,18^. The importance of Mcl1 during development is highlighted by the fact that knockout results in peri-implantation embryonic lethality^19^. Mcl1 is required for proper B- and T-cell maturation^20^, whereas various malignancies display elevated levels of Mcl1^21^.

A handful of previous studies have suggested that various members of the Bcl2 family contribute to survival or cell death of dopaminergic neurons. For example, knockdown of Bax or Bak increases the viability of SH-SY5Y dopaminergic cells in response to apoptotic stress, whereas knockdown of Mcl1, Bcl2, or Bcl1-xL decreases viability^22^. Transgenic ectopic expression of Bcl2 itself under the control of the *Th* promoter suggested that the Bcl2 family plays a positive role in controlling the number of dopaminergic neurons present after birth as well as determining the resilience towards apoptotic stressors^5^.

More recently, Mcl1 levels were shown to be altered in MPTP-treated mice. 24 h after the initial MPTP injections there was an expected marked reduction in dopamine terminals in the striatum as well as a drop in the amount of Th and dopamine transporter positive neurons. Whereas the amount of Bax remained statistically unchanged, the levels of Mcl1 were reduced by roughly 40%. The authors suggest that Mcl1 degradation precedes Bax activation and the apoptotic phenotype^23^. Although suggestive, the functional contribution of Mcl1 as a protective dopaminergic cell survival factor was not investigated. Contrary to the previous study, MPTP has been shown to lead to an upregulation of Mcl1 at the messenger RNA and protein level in SH-SY5Y cells suggesting a compensatory mechanism^24^.

Most previous studies of the Bcl2 family in the nervous system used either knockdown strategies or overexpression techniques, possibly allowing secondary effects or compensation to occur. Since small molecule Bcl2 family inhibitors act rapidly at a specific binding site of the target and result in near complete blockade of a particular protein-protein interface, they are ideally suited to identify the most pivotal survival factors, circumventing the drawbacks of genetic techniques^21^. We employed a panel of small-molecule inhibitors of Bcl2 function developed by the pharmaceutical industry for use in oncology which allowed us to pinpoint Mcl1 as a key pro-survival Bcl2 factor in dopaminergic neurons. Importantly, the inhibitors which specifically interfere with the pro-survival function of Mcl1 had lesser effect on a non-dopaminergic cell line, indicating that the inhibitors are not toxic to every cell-type, but display specificity (Figs. 1b, c and 2). Strikingly, UMI-77, a potent Mcl1 inhibitor (with an IC_50_ of 490 nM) rapidly and dose-dependently induced the mitochondrial-dependent apoptotic pathway in MN9D cells, but not N2A cells (Fig. 2g). Western blotting indicates that N2A cell express low levels of Mcl1 as compared to MN9D cell, potentially explaining N2A’s relative resistance to UMI-77 treatment (Fig. 2e). Contrary to Mcl1, the pro-survival Bcl2 factor Bcl-xL is comparably expressed in MN9D cells as well as N2A cells. Inhibition of Bcl-xL with ABT-263 resulted in apoptotic cell death of N2A, but spared MN9D cells.

The specificity of these small molecule Bcl2 inhibitors as well as the differential expression of Bcl2 factors in different cell types suggest that every cell type has a differently calibrated survival system (“Bcl2 code”) which enables precise control of apoptosis during development, but reveals weak links with age and stress^2^. Intriguingly, molecular weights of Mcl1 as well as Bcl-xL appeared to differ between cell types suggesting that besides expression levels, post translational modifications of Bcl2 protein family members also have a cell type-specific signature (Fig. 2e).

### Targeting Mcl1 as a novel therapeutic approach in the treatment of PD?

As discussed above, different cell types express different Bcl2 factors in addition to a cell-specific post-translational modification signature which could explain why dopaminergic neurons are more susceptible to stress in comparison to other cell types. The presence and importance of Mcl1 in dopaminergic neurons might be exploited to enhance the resilience of dopaminergic neurons to stress as is apparent from our described results. But what is the best strategy to enhance Mcl1 function? Mcl1 is a relatively short-lived protein, prone to various post-translational modifications such as phosphorylation and ubiquitination^14^. Interfering with the degradation of Mcl1 may prove a promising strategy. Interestingly, the Bax E3 ligase Parkin has also been reported to target Mcl1^22^, implicating Parkin in controlling the balance between neuronal survival and cell death. How familial mutations in Parkin would shift the balance between this pro-apoptotic and pro-survival Bcl2 factor remains to be studied as well as if these results are the consequence of different cell types studied. More importantly, several additional E3 ubiquitin ligases have been identified that can tag Mcl1 for degradation, which includes Mcl-1 ubiquitin ligase E3 (Mule; also known as Huwe1 or ARF-BP1), Trim17, beta transducin-containing protein (β-TrCP), F-Box and WD containing 7 protein (FBW7), and cell-division cycle protein 20 activated anaphase promoting complex (APC/C^CDC20^)^25–29^. Conversely, ubiquitination of Mcl1 can be reversed by the deubiquitinase USP9X and the more recently identified USP13^30,31^. The expanding list of potential Mcl1 E3 ligases emphasize the need to delve deeper into ubiquitination and degradation of Mcl1 in the dopamine system. Expression of any E3 ligase acting on Mcl1 specifically in the dopamine system would justify pursuing the development of small molecule inhibitors targeting this E3 ligase.

**Fig. 7:**
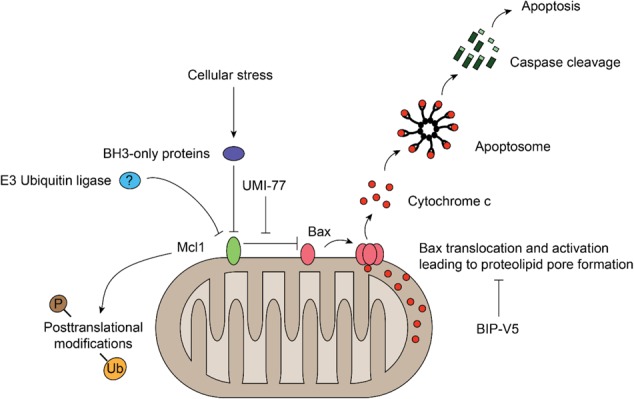
Schematic model depicting the role of Mcl1 in dopaminergic survival. Mcl1 blocks the activation of Bax and thereby prevents mitochondrial outer membrane permeabilization, cytochrome *C* release, and cleaved caspase 3 activation. Possibly regulation of Mcl1 by post-translational modifications can be exploited to therapeutically intervene with cell death occurring in dopaminergic neurons

In conclusion, we have identified Mcl1 as a crucial Bcl2 factor essential for the survival of dopaminergic neurons. The mRNA and protein levels of Mcl1 as well as functional activity all contribute to dopaminergic resilience and may constitute the weakest link in the chain controlling dopaminergic survival (Fig. 7). Enhanced expression or functional protein activity of Mcl1 warrants a focused program of therapeutic development to attenuate dopamine neuron death in response to apoptotic stressors in Parkinson’s disease. Further, the repertoire of Bcl2 factors (“Bcl2 code”) and other apoptosis regulators appears to be uniquely balanced in different cell types beyond the CNS – opening the door to broader cell type-specific ablation or protection strategies.

## Material and methods

### Animals

All animal experimentation was supported and granted by the Animals Experimentation Committee of the University of Amsterdam according to national and international legislation. The morning of detection of the vaginal plug was considered as E0.5. Tissue was isolated at post-natal day P2 (day of birth + 2). Animals are cared for on a daily basis according to rules and regulation of the Dutch and European law. Animals were killed according to rules and regulation of Dutch and European law.

### Fluorescence-activated cell sorting (FACS) and dissection

Midbrains and rostral hindbrains of Pitx3GFP/+ heterozygous mice were dissected at P2 in L15-5% Fetal Calf Serum (Gibco). Dissociation and sorting of mid-hindbrains were performed as described previously^7^. In short, freshly isolated tissue were dissociated using a Papain dissociation system (Worthington). Cells were sorted on a BD FACS Aria III using previously described settings^7^ and collected in Trizol-LS (Invitrogen).

### Quantitative PCR

Relative expression levels were determined by quantitative PCR (qPCR) real-time qPCR (Lightcycler 480) using the QuantiTect SYBR Green RT PCR Kit (QIAGEN) according to the manufacturer’s instructions. For each reaction an estimated 0.1 ng (FACsorted neurons) total RNA was used as input. Primer sequences used were: *Mcl1* forward 5′-*CGTAACAAACTGGGGCAGGAT*-3′; *Mcl1* reverse 5′-*CAAACCCATCCCAGCCTCTTT*-3′; *Bcl2* forward 5′-*ACGGAGGCTGGGATGCCTTTG-*3′; *Bcl2* reverse 5′-*AGTGATGCAGGCCCCGACCA*-3′; *Bcl-xL* forward 5′-*CGGGGCACTGTGCGTGGAAA-*3′; *Bcl-xL* reverse 5′-*AAGTGTCCCAGCCGCCGTTC*-3′; *Bax* forward 5′-*TGGCAACTTCAACTGGGGCCG*-3′; *Bax* reverse 5′-*AGAGGAGGCCTTCCCAGCCAC*-3′. Amplification products were separated on an agarose gel to confirm product size. Data was analyzed using the ΔΔCt method. Primers were assumed to have comparable efficiency.

### Chemical inhibitors and stressors

UMI-77 (APExBIO);^8^ Bax-inhibiting peptide V5 (BIP-V5; Sigma Aldrich); MG132 (Enzo Life Sciences), etoposide (Cell Signaling Technologies); PAC1, Bam7, Birinapant, Lexibulin (CYT997), Nutlin-3a, YM155, A1210477, Navitoclax (ABT-263), Venetoclax (ABT-199) and Obatoclax mesylate were all purchased from Selleckchem. All inhibitors and stressors were dissolved in DMSO; control conditions were all treated with the appropriate amount of DMSO.

### Cell culture and transfections

MN9D cells were cultured on poly-d-lysine (Sigma-Aldrich) coated culture dishes in Dulbecco’s Modified Eagle’s Medium (DMEM; Lonza) supplemented with 200 nM l-glutamine (ThermoFisher Scientific), 1x Penicillin/Streptomycin (Pen/Strep; 100 units/ml; ThermoFisher Scientific) and 10% heat inactivated fetal bovine serum (HiFBS; Biowest). Neuroblastoma 2 A cells were cultured in the same medium. Cells were incubated in a 5% CO_2_ / 95% O_2_ atmosphere at 37 °C. A day before the inhibitor and/or stressor experiments, cells were serum deprived in DMEM containing 200 nM l-glutamine, 1x Pen/Strep and 0.5% HiFBS to limit growth factor interference. Transfections were performed with lipofectamine 2000 (Invitrogen) according to manufacturer’s instructions.

### Cleaved caspase-3 assay

After treatment with the indicated inhibitors, cells were lysed with 0.1% Triton-X100 (Sigma-Aldrich) and incubated with 10 µM AC-DEVD-AMC fluorogenic cleaved caspase-3 (CC3) substrate (ALX-260-031; Enzo Life Sciences) for 30 min. Fluorescence was measured at an excitation wavelength of 350 nm and emission at 450 nm using a BioTek Synergy H1 plate reader.

### Western blotting

After treatment, cells were washed with cold phosphate buffered saline (PBS; Lonza) and lysed in 1x Laemmli sample buffer (62.5 mM Tris-HCl pH 6.8, 2% [SDS; Merck Millipore], 10% glycerol [Sigma-Aldrich] and 0.01% w/v bromophenol blue [Sigma-Aldrich] in MilliQ water supplemented with 50 mM dithiotretol [DTT; Merck Millipore]). Samples were collected, sonicated for 3 min in a Bioruptor sonicator (Diagenode) on HIGH settings and boiled at 95 °C for 5 min. Equal amounts of protein were loaded and separated on a SDS-PAGE gel (10–14% depending on molecular weight), followed by transfer to Amersham Protran 0.2 µm nitrocellulose membranes (GE Healthcare Life Sciences). Subsequently, membranes were blocked in 5% ELK milkpowder (Campina) in Tris-buffered saline-0.1% Tween-20 (TBS-T). Blots were incubated with the appropriate primary antibody in TBS-T overnight at 4 °C. Blots were incubated with goat-anti-rabbit Mcl1 (#94296; 1:1000), goat-anti-rabbit Bcl-xL (#2764; 1:1000), goat-anti-mouse β-actin (#3700; 1:5000), goat-anti-rabbit Bax (#14796; 1:1000), and goat-anti-rabbit CC3 (#9664; 1:1000). All antibodies were purchased from Cell Signaling Technologies. After incubation with the appropriate HRP-conjugated secondary antibody (Invitrogen) in TBS-T, the blots were exposed to enhanced chemiluminescence (ECL) reagents. ECL reagents contained 250 mM luminol (Fluka Analytical), 90 mM p-coumaric acid (Sigma-Aldrich), 1 M Tris-HCl pH 8.5 and 0.02% H_2_O_2_. Blots were detected on an Odyssey FC Imaging System (LI-COR Biosciences). Densitometric analyses were performed in ImageStudio Lite software (LI-COR Biosciences). Differences in signal between the conditions were tested for significance with a Student's *t*-test. Results were considered significant at *p* < 0.05.

### Immunofluorescence & propidium iodide staining

For immunofluorescence experiments, MN9D cells were grown on poly-D-lysine coated coverslips. Cells were then serum deprived as described above and treated with the appropriate inhibitors/peptides. After treatment, cells were fixed for 10 min in 4% paraformaldehyde (PFA; Merck Millipore). Permeabilization was performed in 0.01% Triton X-100 in PBS for 30 min, followed by PBS washes and blocking in 3% bovine serum albumin (BSA; Sigma-Aldrich) and 0.01% Triton X-100 in PBS for 1 h at room temperature. Subsequently, cells were incubated overnight with goat-anti-mouse Bax 6A7 (Santa Cruz sc-23959; 1:200) and goat-anti-rabbit CC3 (Cell Signaling Technologies #9664; 1:200) at 4 °C. After PBS washes, cells were incubated with goat-anti-mouse Alexa 555 and goat-anti-rabbit Alexa 488 secondary antibodies (ThermoFisher Scientific), respectively. Following PBS washes, nuclei were stained with DAPI (1:3000) and coverslips were embedded in Fluorsave Reagent (Merck Millipore). For propidium iodide (PI) experiments, cells were grown on poly-D-lysine coated coverslips in 12-wells plates, treated with the appropriate inhibitor, washed with cold PBS and immediately stained with PI (1:1000) for microscopic analysis. Images were obtained with a Leica DM5500 B microscope. The immunostained cells were analyzed using ImageJ software. Differences between conditions were tested for significance with a Student's *t*-test. Results were considered significant at *p* < 0.05.

### Ex vivo slice culture & free-floating immunohistochemistry

Adult Pitx3GFP/ + heterozygous or wildtype mice (1 month old) brains were freshly isolated and sliced on a Leica VT100S vibratome in icecold PBS. Midbrain slices with a thickness of 250 µm were obtained and checked for GFP expression in case of the Pitx3GFP/ + mice. Subsequently, the slices were divided in half to have an internal control. Slices were cultured overnight in explant culture medium as described previously^9^ in the presence of either DMSO or 10 µm UMI-77 in a 5% CO_2_/95% O_2_ atmosphere at 37 °C. The next day, slices were briefly washed in PBS and fixed in 4% PFA for 30 min at 4 °C. After fixation, the slices were briefly washed in PBS followed by blocking in 3% BSA, 0.5% Triton-X in PBS for 6 h at 4 °C. Subsequently slices of Pitx3GFP/ + origin were incubated with goat-anti-chicken GFP (1:500; Abcam) and goat-anti-rabbit CC3 (1:200; CST) in PBS-T, whereas slices of wildtype mice were incubated with donkey-anti-sheep Th (1:500; Abcam) and goat-anti-rabbit CC3 (1:200; CST) in PBS-T overnight at 4 °C. The next day, the slices were washed for 6 times 15 min in PBS-T and incubated with goat-anti-chicken Alexa 488 (1:500) and goat-anti-rabbit Alexa 555 (1:500) or alternatively with donkey-anti-sheep Alexa 488 (1:500) and goat-anti-rabbit Alexa 555 (1:500) for 4 h at RT. Following secondary antibody incubation, the slices were washed 4 times for 15 min and were incubated with DAPI (1:3000) to stain nuclei. Finally, slices were embedded on microscopy slides in Fluorsave reagent.

### Confocal microscopy

Confocal images of the immunostained *substantia nigra* were obtained using a Nikon A1 confocal microscope. The following fluorphores were visualized with the following settings (excitation/emission): DAPI 405 nm/425–475 nm), eGFP (488 nm/500–550 nm) and Alexa 594 (594 nm /570–620 nm). Z-stack images were taken using a 20x objective. Additional settings: pinhole 0.7, scan size 1024, speed 0.125, and average 2. Image analysis was performed using ImageJ software (Supplementary Fig. 1). Differences between conditions were tested for significance with a Student's *t*-test. Results were considered significant at *p* < 0.05.

## Electronic supplementary material

figure S1

supplementary figure legends
