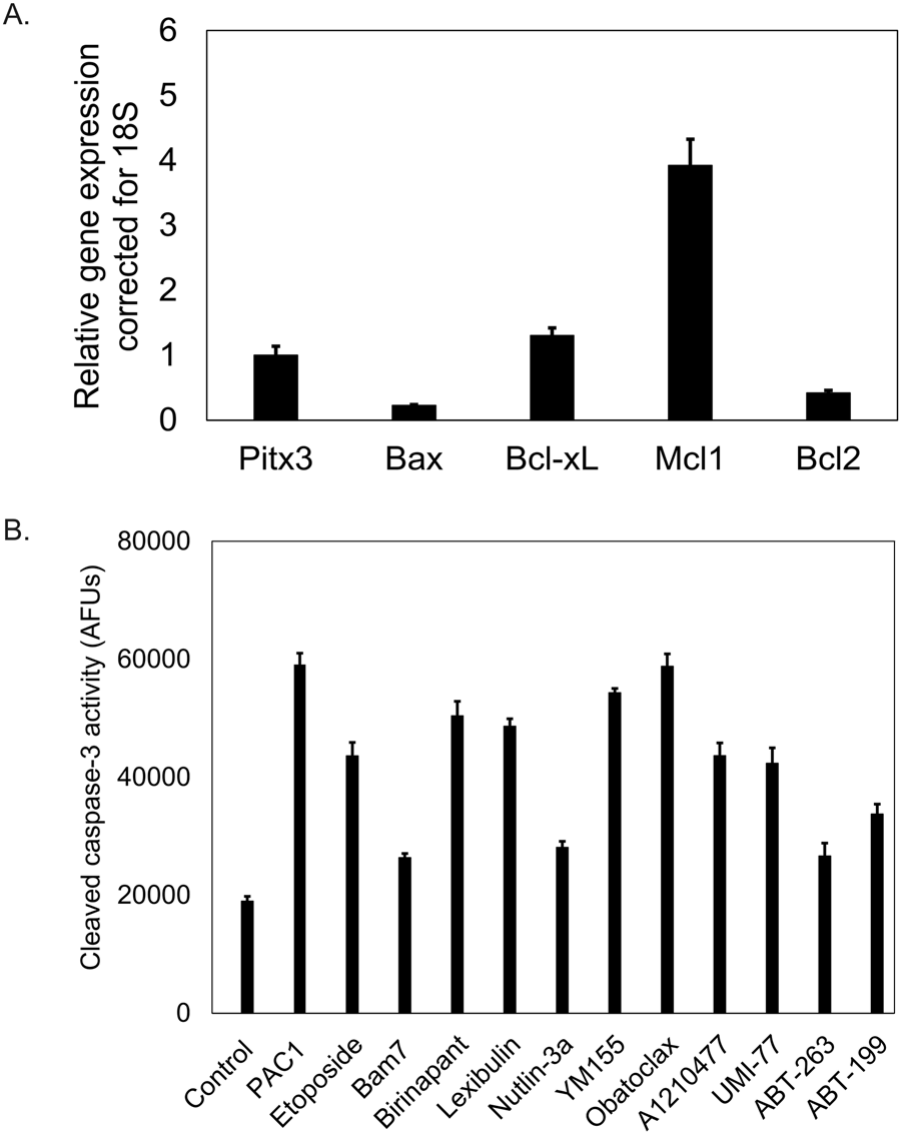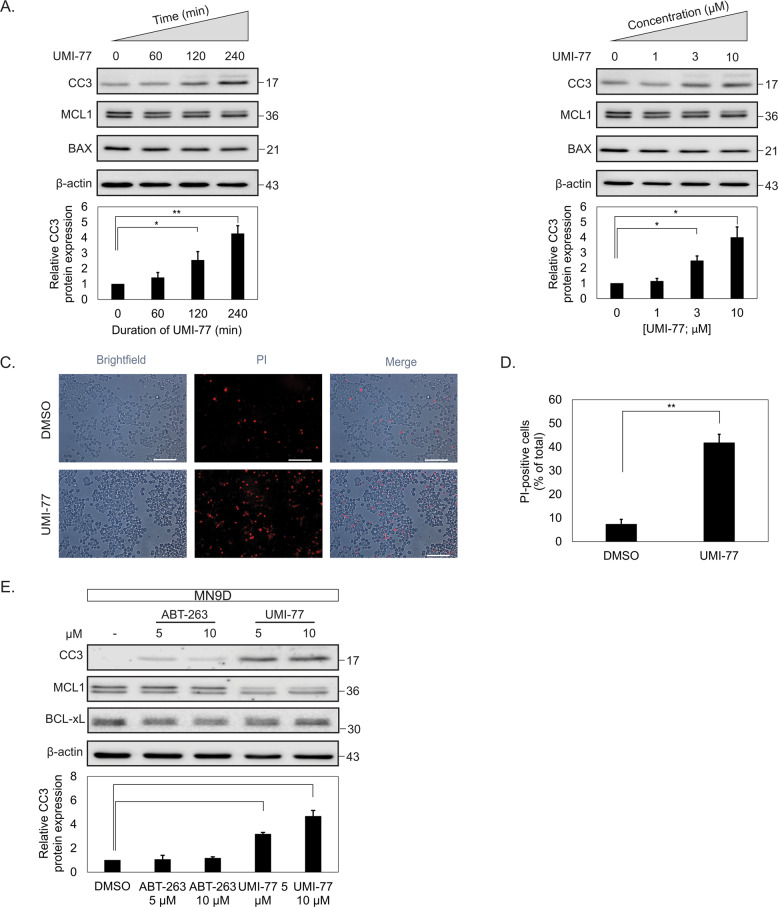# Correction to: Survival of midbrain dopamine neurons depends on the Bcl2 factor Mcl1

**DOI:** 10.1038/s41420-022-00871-3

**Published:** 2022-03-07

**Authors:** Edward J. Robinson, Sebastian P. Aguiar, Willemieke M. Kouwenhoven, Dorinde S. Starmans, Lars von Oerthel, Marten P. Smidt, Lars P. van der Heide

**Affiliations:** Swammerdam Institute for Life Sciences, University of Amsterdam, Science Park 904, 1098 XH Amsterdam, The Netherlands

Correction to: *Cell Death Discovery* 10.1038/s41420-018-0125-7, published online 21 November 2018

The original version of this article unfortunately contained mistakes in Figs. 1 and 2. Preliminary experiments with a new batch of neuro2a cells from atcc suggests that neuro2a cells behave similarly to the mn9d cells used in the manuscript (they are sensitive to Mcl1 inhibition with umi-77 and Mcl1 appears to have the same molecular weight as in mn9d cells). The corrected figures can be found below.