# Supplementary figures and images for: Survival of midbrain dopamine neurons depends on the Bcl2 factor Mcl1
Source: Cell Death Discov. 2018 Nov 21;4:107. doi: 10.1038/s41420-018-0125-7 (PMC6249233; doi:10.1038/s41420-018-0125-7)

Fig S1 – Counting procedure

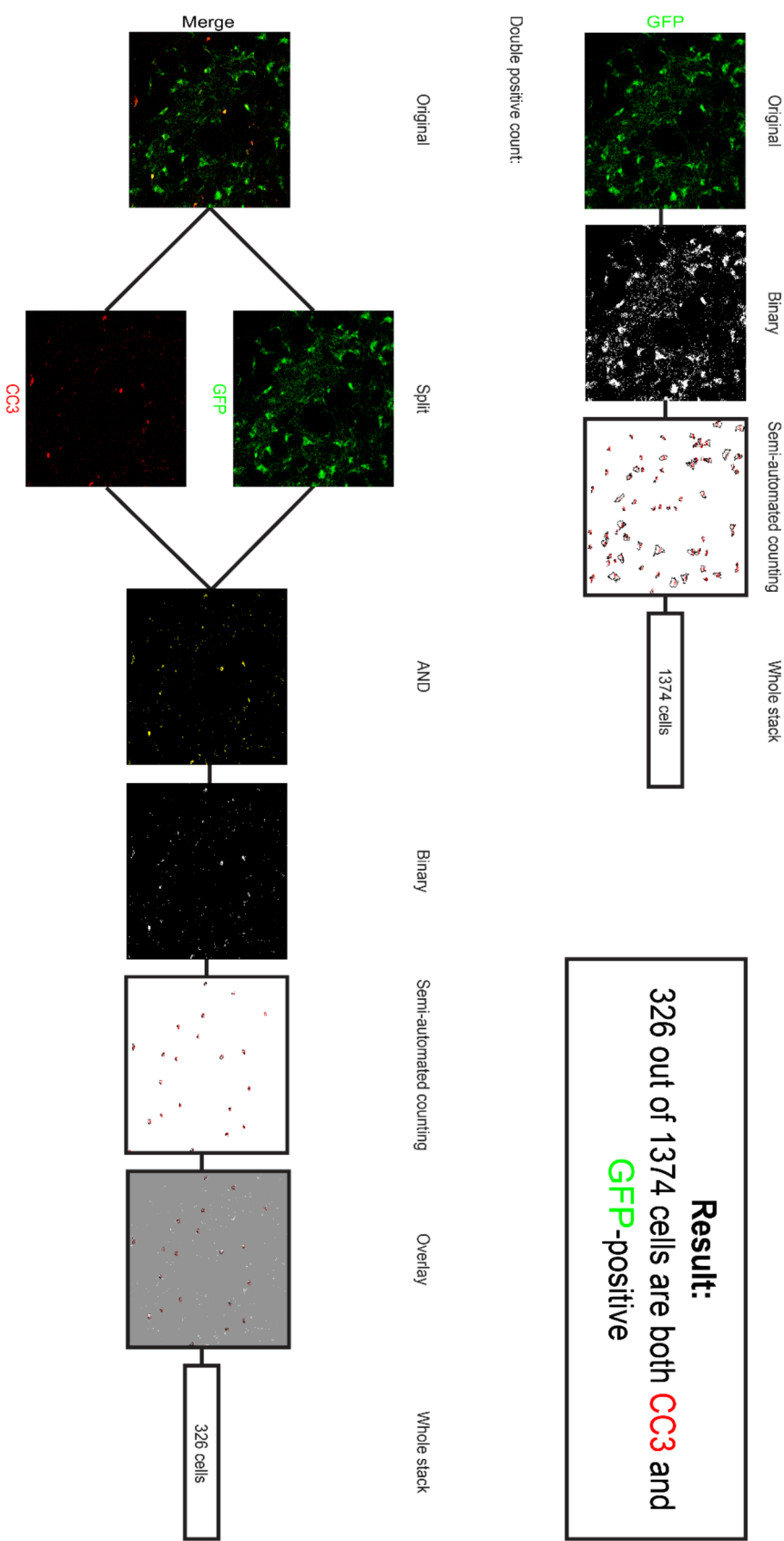

Supplement: Supplementary file 1 — figure S1 [file 41420_2018_125_MOESM1_ESM.pdf]
