# Supplementary material for: Survival of midbrain dopamine neurons depends on the Bcl2 factor Mcl1
Source: Cell Death Discov. 2018 Nov 21;4:107. doi: 10.1038/s41420-018-0125-7 (PMC6249233; doi:10.1038/s41420-018-0125-7)
Supplement: Supplementary file 2 — supplementary figure legends [file 41420_2018_125_MOESM2_ESM.docx]

**Figure S1**. Quantification of stained cells in *ex vivo* slice cultures. Confocal images of an overlay of the channels were imported into ImageJ and subsequently split to obtain the individual channels. The ImageJ function AND (Process > Image Calculator > Select two images > AND) was used to calculate locations which are both GFP and cleaved caspase 3 positive. This image was converted to a binary image (Process > Binary > Make Binary) and the locations were counted with Analyze Particles (Analyze > Analyze Particles) and showing the outlines of counted cells. Finally, the output image was overlayed with the original image to check if indeed only cells were counted.
